# Identifying Patients With Heart Failure Who Are Susceptible to De Novo Acute Kidney Injury: Machine Learning Approach

**DOI:** 10.2196/37484

**Published:** 2022-10-14

**Authors:** Caogen Hong, Zhoujian Sun, Yuzhe Hao, Zhanghuiya Dong, Zhaodan Gu, Zhengxing Huang

**Affiliations:** 1 College of Biomedical Engineering and Instrument Science Zhejiang University Hangzhou China; 2 Jiangsu Automation Research Institute Lianyungang China; 3 Research Center for Applied Mathematics and Machine Intelligence Zhejiang Lab Hangzhou China; 4 College of Computer Science and Technology Zhejiang University Hangzhou China

**Keywords:** heart failure, acute kidney injury, unsupervised machine learning, risk stratification, phenogrouping

## Abstract

**Background:**

Studies have shown that more than half of patients with heart failure (HF) with acute kidney injury (AKI) have newonset AKI, and renal function evaluation markers such as estimated glomerular filtration rate are usually not repeatedly tested during the hospitalization. As an independent risk factor, delayed AKI recognition has been shown to be associated with the adverse events of patients with HF, such as chronic kidney disease and death.

**Objective:**

The aim of this study is to develop and assess of an unsupervised machine learning model that identifies patients with HF and normal renal function but who are susceptible to de novo AKI.

**Methods:**

We analyzed an electronic health record data set that included 5075 patients admitted for HF with normal renal function, from which 2 phenogroups were categorized using an unsupervised machine learning algorithm called K-means clustering. We then determined whether the inferred phenogroup index had the potential to be an essential risk indicator by conducting survival analysis, AKI prediction, and the hazard ratio test.

**Results:**

The AKI incidence rate in the generated phenogroup 2 was significantly higher than that in phenogroup 1 (group 1: 106/2823, 3.75%; group 2: 259/2252, 11.50%; *P*<.001). The survival rate of phenogroup 2 was consistently lower than that of phenogroup 1 (*P*<.005). According to logistic regression, the univariate model using the phenogroup index achieved promising performance in AKI prediction (sensitivity 0.710). The generated phenogroup index was also significant in serving as a risk indicator for AKI (hazard ratio 3.20, 95% CI 2.55-4.01). Consistent results were yielded by applying the proposed model on an external validation data set extracted from Medical Information Mart for Intensive Care (MIMIC) III pertaining to 1006 patients with HF and normal renal function.

**Conclusions:**

According to a machine learning analysis on electronic health record data, patients with HF who had normal renal function were clustered into separate phenogroups associated with different risk levels of de novo AKI. Our investigation suggests that using machine learning can facilitate patient phengrouping and stratification in clinical settings where the identification of high-risk patients has been challenging.

## Introduction

Acute kidney injury (AKI) is a common disorder in patients with heart failure (HF), with the reported incidence rate varying from 7% to 38% in cardiology departments [1-3]. A recently conducted nationwide survey in China showed that about 85% of AKI incidents that occurred during cardiac hospitalization were ignored or were late to be identified [4,5]. As an independent risk factor, the delayed recognition of AKI has been proven to be associated with worse outcomes of patients with HF (eg, chronic kidney diseaseand mortality) [4,6]. To this end, the prompt identification of patients with HF at high-risk of AKI has great potential to improve clinical outcomes.

Although a few specific clinical markers (eg, estimated glomerular filtration rate [eGFR]) have been adopted to evaluate the renal function of patients with HF such that those at high risk of AKI can be identified, these markers lack the ability to screen de novo AKI patients who had normal renal function at admission [7,8]. Of note, several recently conducted population studies have indicated that more than half of the AKI that occurred in patients with HF were de novo [1-3]. To address this challenge, we attempted to clarify the characteristics of patients with HF who are susceptible to de novo AKI and developed a machine learning model for identification of HF patients with normal renal function but at high risk of de novo AKI.

As recently conducted cardiovascular studies have demonstrated that an unsupervised machine learning approach is able to model correlations among variables that contain prognostic information and cluster cohesive patients into 1 homogeneous phenogroup [9-11], we hypothesized that it can also be applied to identify patients with HF at high risk of de novo AKI. Recently, with the rapid development of hospital information systems, a large collection of electronic health records (EHRs) has become available that documents various types of patient information (eg, vital signs, laboratory test results) and treatments (eg, medication, surgery) and thus offers the considerable potential to implement a large-scale real-world analysis at a low expenditure. Therefore, in this study, we aimed to develop an EHR-based unsupervised machine learning analysis to group patients with HF and identify those who are susceptible to de novo AKI.

## Methods

### Study Population

**Figure 1:**
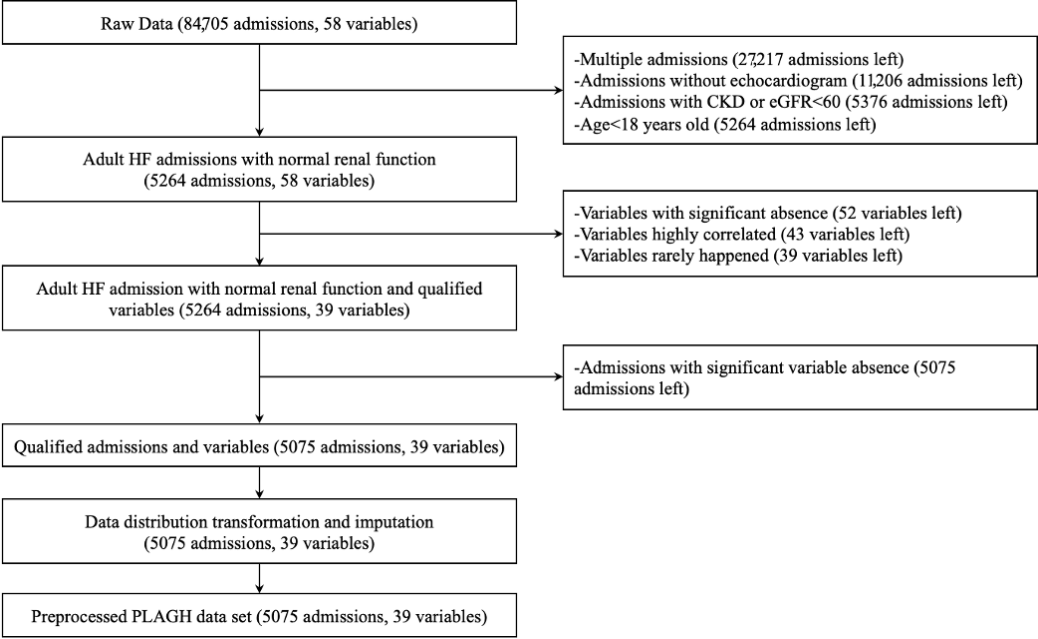
Preprocessing procedure of the PLAGH data set. CKD: chronic kidney disease; eGFR: estimate glomerular filtration rate; HF: heart failure; PLAGH: PLA General Hospital.

The proposed retrospective study used a real-world data set obtained from the EHR system of the Chinese PLA General Hospital (PLAGH). The data set documented regular medical information in 84,705 hospitalizations of 29,699 patients who were diagnosed with HF in the PLAGH from 1998 to 2018. Adult patients with HF and normal renal function (eGFR >60 mL/min/1.73m^2^ as calculated by the serum creatinine [SCr] version of the Chronic Kidney Disease Epidemiology Collaboration [CKD-EPI] equation [12] and without chronic kidney disease diagnosis) were considered for inclusion. Additionally, patients who did not have echocardiogram records were excluded. For patients with multiple hospitalizations, only the last hospitalization was reserved. The detailed preprocessing procedure is illustrated in Figure 1.

### Ethics Approval

The study protocol was approved, with a waiver of consent granted on the basis of minimal harm and general impracticability by the health institutional review board of Zhejiang University (No. ZJU-2021-27).

### Variable Selection and Machine Learning Model

In this study, 58 variables potentially associated with AKI, including demographics, vital sign measurements, medications, laboratories, operations, and echocardiogram exams, and routinely documented in EHRs at the admission stage of hospitalization were considered as candidates for analysis. To ensure that the most informative variables were selected and the correlation between variables could be diluted, we excluded variables with a missing rate larger than 30% or with a Pearson correlation coefficient >0.6 or that were documented fewer than 100 times in the raw EHR data set. As a result, 39 variables were included in the cohort. All continuous variables were transformed to standard normal distribution for the convenience of the unsupervised machine learning model (Table S1, Multimedia Appendix 1). Thereafter, we adopted multivariate imputation by chained equations [13] to impute the missing data.

We employed a simple yet effective unsupervised machine learning model called K-means clustering to categorize patients into different phenogroups [14]. The silhouette coefficient was applied to determine the optimal number of phenogroups [15]. We also adopted the nonlinear dimensionality reduction technique of t-distributed stochastic neighbor embedding [16] to visualize and evaluate the clustering results in a qualitative manner. The model was repeatedly run 1000 times to guarantee the achieved results stable.

### Outcomes of Interest

The primary outcome was the incidence of AKI, which was defined according to the Kidney Disease: Improving Global Outcomes (KDIGO) standard [17], with the occurrence of AKI defined as the increase of SCr to ≥1.5 times the baseline in 7 days or the increase of SCr by ≥26.5 μmol/L within 48 hours. The secondary outcome was in-hospital mortality.

### Characterization of Phenogroups

Once patients with HF were categorized into separate phenogroups, we measured the differences of variables in different groups. Continuous variables are reported as median and IQR (interquartile range). Categorical variables are reported as the frequencies and counts. Differences between groups were tested using the 1-way analysis of variance, Kruskal-Wallis test, or the chi-square test where appropriate. A *P* value of <.01 was considered statistically significant.

### Discrimination of Phenogroups

We validated whether the phenogroup index generated by K-means clustering correlated with outcomes of interests by carrying out the following 3 experiments. First, Kaplan-Meier estimators with log-rank tests were conducted to analyze the time-to-event characteristics in different phenogroups. Second, we compared the prediction performance on AKI and in-hospital mortality to check whether the inferred phenogroup index was an effective risk predictor for outcomes of interest. Specifically, we selected the top-ranked 10 variables using a forward stepwise strategy with the Akaike information criterion and then developed 5 logistic regression (LR) models to predict the outcomes of interest. Model 1 used the phenogroup index as the univariate predictor. Model 2 used the top-ranked 10 variables as predictors. Model 3 used the top-ranked 10 variables and the phenogroup index. Model 4 used all 39 variables. Model 5 used all 39 variables and the phenogroup index. All models were trained by 70% of the data from the PLAGH data set and tested with the remaining 30% of data. Third, to evaluate whether the phenogroup index could achieve the competitive discriminative performance compared to the original variables with respect to the primary and secondary outcomes, we applied unadjusted Cox proportional hazard regression to examine hazard ratios (HRs), 95% CIs, and *P* values for all included original variables as well as the phenogroup index on both the whole PLAGH data set and the following subgroups: age (age <65 vs ≥65 years), sex, type of HF (acute vs chronic), diabetes mellitus, stroke, atrial fibrillation, coronary heart disease, anemia, and left ventricular ejection fraction (<40%, 40%-49%, and ≥50%). To assess continuous variables appropriately, we categorized all continuous variables in validation, and the cutoff points for these continuous variables are presented in online supplementary Table S2, Multimedia Appendix 1.

### External Validation

We externally validated our model on a well-known open-source database, Medical Information Mart for Intensive Care (MIMIC)-III [18]. After a requisite preprocessing procedure (online supplementary, Figure S1), we prepared a MIMIC-III data set that contained 1006 patients with HF who had normal renal function. The model trained by the PLAGH data set was directly transferred onto the MIMIC-III data set. In detail, we compared the distance between the data of each patient in the MIMIC-III data set and the centroids of the derived phenogroups from the PLAGH data set and then assigned the patient into a phenogroup with the minimum Euclidean distance. After that, we assessed the survival rate and prediction performance of AKI and in-hospital mortality of the generated phenogroups from the MIMIC-III data set. As patients contained in the PLAGH data set were mainly from general wards in the PLAGH and patients included in the MIMIC-III data set were from intensive care units in the United States, there inevitably were statistical differences between the baseline characteristics of patients in the 2 data sets (Table S3, Multimedia Appendix 1). In this sense, the external validation was able to evaluate the stability of the proposed model in diverse clinical settings.

In this study, statistical and machine learning analysis was based on sklearn, lifelines, scipy package [19-21], and Python. We also report the centroids of the generated phenogroups from the PLAGH data set (Table S4, Multimedia Appendix 1), which may be nontrivial knowledge to assist clinicians in identifying their patients with HF at high risk of de novo AKI.

## Results

### Phenogroup Results

After preprocessing, 5075 hospitalizations and 39 variables (Table 1) were reserved for the PLAGH data set (median age 61 years, IQR 51-70 years; female 1723/5075, 32.39%; acute HF 1723/5075, 33.95%). Using K-means clustering, we naturally separated patients into 2 basically nonoverlapping phenogroups, where the number of clusters was suggested by the silhouette coefficient test (Figure S1, Multimedia Appendix 1). Similar results were found using t-distributed stochastic neighbor embedding visualization (Figure S2, Multimedia Appendix 1).

**Table 1:** Included variables for clustering.

Domain	Features
Demographic	Age, sex
Disease	Acute/chronic HF, atrial fibrillation, cardiomyopathy, coronary heart disease, diabetes, stroke, valvular heart disease
Medication	Angiotensin-converting enzyme inhibitor/angiotensin receptor blocker, anticoagulant, antiplatelet, beta blocker, calcium channel blocker, diuretic, positive inotropic drug, vasodilator
Echocardiography	Left ventricular ejection function
Laboratory result	Alanine aminotransferase, aspartate transaminase, estimated glomerular filtration rate, gamma-glutamyl transferase, hemoglobin, high-density lipoprotein cholesterol, low-density lipoprotein cholesterol, N-terminal probrain natriuretic peptide, serum calcium, serum potassium, serum sodium, serum urea, total bilirubin, total serum protein, triglyceride, troponin T
Operation	Angiography percutaneous coronary intervention
Vital sign	BMI, diastolic blood pressure, systolic blood pressure

^a^Only drugs used in the first 48 hours after admission were included to ensure the drug usage could reflect the patient admission status.

### Characteristics of Phenogroups

Table 2 illustrates the baseline characteristics of the PLAGH data set and the 2 derived phenogroups. Compared to phenogroup 1, phenogroup 2 had a higher rates of AKI (group 1: 106/2823, 3.75%; group 2: 259/2252, 11.50%; *P*<.001) and in-hospital mortality (phenogroup 1: 21/2823, 0.74%; phenogroup 2: 118/2252, 5.24%; *P*<.001). In addition, patients in phenogroup 2 were generally older than those in phenogroup 1 (58 vs 65 years; *P*<.001).

As can be seen in Table 2, there are more patients diagnosed with acute HF in phenogroup 2 than those in phenogroup 1 (phenogroup 1: 738/2823, 26.14%; phenogroup 2: 985/2252, 43.74%; *P*<.001). Moreover, cardiac function of patients in phenogroup 2 was worse than that in phenogroup 1. Specifically, there were statistical differences between patients in phenogroup 1 and phenogroup 2 in terms of left ventricular ejection fraction (50% vs 41%; *P*<.001), diastolic blood pressure (77 mmHg vs 70 mmHg; *P*<.001), systolic blood pressure (130 mmHg vs 118 mmHg; *P*<.001), N-terminal pro-brain natriuretic peptide (572 pg/mL vs 2680 pg/mL; *P*<.001), hemoglobin (143 g/L vs 129 g/L; *P*<.001), atrial fibrillation (phenogroup 1: 526/2823, 18.63%; phenogroup 2: 595/2252, 26.42%; *P*<.001), diuretic usage (phenogroup 1: 1608/2823, 56.96%; phenogroup 2: 1799/2252, 79.88%; *P*<.001), and positive inotropic drug usage (phenogroup 1: 778/2823, 27.56%; phenogroup 2: 1089/2252, 48.36%; *P*<.001). Furthermore, phenogroup 2 had higher troponin T levels (0.01 ng/mL vs 0.02 ng/mL; *P*<.001), indicating that there were more patients in phenogroup 2 who underwent myocardial damage. Patients in phenogroup 2 had higher values of gamma-glutamyl transferase (31.70 IU/L vs 40.30 IU/L; *P*<.001), total bilirubin (12.79 μmol/L vs 15.85 μmol/L; *P*<.001), and aspartate aminotransferase (19.60 IU/L vs 24.29 IU/L; *P*<.001), indicating that patients in phenogroup 2 might have worse liver function compared with phenogroup 1. Moreover, although we had excluded patients with renal dysfunction in advance, patients in phenogroup 2 had worse eGFR values (92.06 mL/min/1.73m^2^ vs 81.85 mL/min/1.73 m^2^; *P*<.001) and urea (5.46 mmol/L vs mmol/L; *P*<.001). These findings demonstrated that patients in phenogroup 2 had relatively worse kidney function. Furthermore, patients in phenogroup 2 used less angiotensin-converting enzyme inhibitor/angiotensin receptor blocker (phenogroup 1: 1531/2823, 54.23%; phenogroup 2: 1016/2252, 45.11%; *P*<.001), calcium channel blocker (phenogroup 1: 789/2823, 27.95%; phenogroup 2: 321/2252, 14.25%; *P*<.001), and antiplatelets (phenogroup 1: 1914/2823, 67.80%; phenogroup 2: 1384/2823, 61.45%; *P*<.001). It was worth nothing that patients in phenogroup 2 had higher lipid levels (low-density lipoprotein cholesterol and triglyceride) and BMI (25.88 kg/m^2^ vs 23.05 kg/m^2^; *P*<.001). Compared to phenogroup 1, phenogroup 2 also received less angiography (phenogroup 1: 1311/2823, 46.44%; phenogroup 2: 1311/2252, 30.95%; *P*<.001) and percutaneous coronary intervention (phenogroup 1: 640/2823, 21.96%; phenogroup 2: 349/2252, 15.50%; *P*<.001). Comprehensive baseline characteristics including all 58 candidate variables are listed in Table S5, Multimedia Appendix 1.

**Table 2:** Baseline characteristics of the PLA General Hospital data set and the generated phenogroups.

Feature			Population (N=5075)	Phenogroup 1 (n=2823)	Phenogroup 2 (n=2252)	*P* value
**Feature of interest,** **n (%)**						
	AKI^a^		365 (7.19)	106 (3.75)	259 (11.50)	<.001
	In-hospital mortality,		139 (2.74)	21 (0.74)	118 (5.24)	<.001
**Demographic**						
	Age (years), median (IQR)		61 (51-70)	58 (48-67)	65 (55-75)	<.001
	BMI (kg/m^2^), median (IQR)		24.60 (22.46-27.08)	25.88 (23.87-28.08)	23.05 (20.95-25.01)	<.001
	DBP^b^ (mmHg), median (IQR)		74 (67-81)	77 (70-85)	70 (64-78)	<.001
	SBP^c^ (mmHg), median (IQR)		125 (113-138)	130 (119-143)	118 (106-130)	<.001
	Male, n (%)		3431 (67.61)	1943 (68.83)	1488 (66.07)	<.001
**Disease,** **n (%)**						
	**HF^d^**					
		Acute HF	1723 (33.95)	738 (26.14)	985 (43.73%)	<.001
		Chronic HF	3352 (66.05)	2075 (73.86)	1267 (56.26%)	<.001
	AF^e^		1121 (22.09)	526 (18.63)	595 (26.42)	<.001
	Cardiomyopathy		941 (18.54)	497 (17.61)	444 (19.71)	<.001
	CHD^f^		2928 (57.69)	1660 (58.80)	1268 (56.30)	.07
	Diabetes		2002 (39.44)	1041 (36.88)	961 (42.67)	<.001
	Stroke		485 (9.56)	282 (9.99)	233 (10.35)	.09
	VHD^g^		616 (12.13)	336 (11.90)	280 (12.43)	.57
**Medication,** **n (%)**						
	ACEI/ARB^h^		2547 (50.18)	1531 (54.23)	1016 (45.11)	<.001
	Anticoagulant		1927 (37.97)	989 (35.03)	938 (41.65)	<.001
	Antiplatelet		3298 (64.99)	1914 (67.80)	1384 (61.45)	<.001
	Beta blocker		3428 (67.54)	1981 (70.17)	1447 (64.25)	<.001
	CCB^i^		1110 (21.87)	789 (27.95)	321 (14.25)	<.001
	Diuretic		3407 (67.13)	1608 (56.96)	1799 (79.88)	<.001
	Positive inotropic drugs		1867 (36.79)	778 (27.56)	1089 (48.36)	<.001
	Vasodilator		3103 (61.14)	1698 (60.15)	1405 (62.39)	.10
**Echocardiogram**						
	LVEF^j^, median (IQR)		46 (35-56)	50 (39-58)	41 (31-54)	<.001
	<40%, n (%)		1716 (33.81)	719 (25.47)	997 (44.27)	<.001
	40%-50%, n (%)		1174 (23.13)	690 (24.44)	484 (21.49)	.05
	≥50%, n (%)		2185 (42.86)	1414 (50.09)	771 (34.24)	<.001
**Laboratory result, median (IQR)**						
	ALT^k^, (IU/L)		21.00 (14.39-33.79)	20.80 (14.70-31.99)	21.54 (13.80-36.49)	<.001
	AST^l^, (IU/L)		21.29 (16.29-30.50)	19.60 (15.50-26.00)	24.29 (18.09-38.80)	<.001
	Calcium (mmol/L)		2.24 (2.16-2.33)	2.28 (2.21-2.36)	2.19 (2.10-2.27)	<.001
	eGFR^m^ (mL/min/1.73 m^2^)		87.62 (75.65-98.80)	92.06 (80.84-101.91)	81.85 (70.90-92.91)	<.001
	GGT^n (^IU/L)		34.80 (21.90-63.79)	31.70 (21.30-54.89)	40.30 (23.09-75.00)	<.001
	HDL-C^o^ (mmol/L)		1.02 (0.85-1.22)	1.04 (0.88-1.22)	1.01 (0.82-1.22)	<.001
	Hemoglobin, g/L		137 (124-150)	143 (132-154)	129 (116-142)	<.001
	LDL-C^p^ (mmol/L)		2.25 (1.79-2.81)	2.46 (1.96-3.05)	2.04 (1.62-2.48)	<.001
	NT-pro-BNP^q^ (pg/mL)		1216 (422-2950)	572 (225-1319)	2680 (1355-5188)	<.001
	Potassium (mmol/L)		3.89 (3.62-4.17)	3.87 (3.62-4.13)	3.91 (3.61-4.20)	.005
	Sodium (mmol/L)		140.70 (138.10-142.70)	141.30 (139.40-143.20)	139.40 (136.30-142.00)	<.001
	Total bilirubin (μmol/L)		13.69 (9.80-19.90)	12.79 (9.40-17.40)	15.85 (10.39-24.60)	<.001
	Total protein (g/L)		67.5 (63.3-71.8)	69.2 (65.8-73.3)	65.1 (60.4-69.0)	<.001
	Triglyceride (mmol/L)		1.11 (0.82-1.59)	1.34 (0.98-1.87)	0.92 (0.72-1.21)	<.001
	Troponin T (ng/mL)		0.01 (0.01-0.04)	0.01 (0.00-0.02)	0.02 (0.01-0.10)	<.001
	Urea (mmol/L)		5.84 (4.73-7.25)	5.46 (4.51-6.60)	6.45 (5.11-8.12)	<.001
**Operation, n (%)**						
	Angiography		2008 (29.57)	1311 (46.44)	697 (30.95)	<.001
	PCI^r^		969 (19.09)	620 (21.96)	349 (15.50)	<.001

^a^AKI: acuted kidney injury.

^b^DBP: diastolic blood pressure.

^c^SBP: systolic blood pressure.

^d^HF: heart failure.

^e^AF: atrial fibrillation.

^f^CHD: coronary artery disease.

^g^VHD: valvular heart disease.

^h^ACEI/ARB: angiotensin-converting enzyme inhibitor/angiotensin receptor blocker.

^i^CCB: calcium channel blocker.

^j^LVEF: left ventricular ejection fraction.

^k^ALT: alanine aminotransferase.

^l^AST: aspartate transaminase.

^m^eGFR: estimated glomerular filtration rate.

^n^GGT: gamma-glutamyl transferase.

^o^HDL-C: high-density lipoprotein cholesterol.

^p^LDL-C: low-density lipoprotein cholesterol.

^q^NT-pro-BNP: N-terminal probrain natriuretic peptide.

^r^PCI: percutaneous coronary intervention.

### Survival Analysis

As the prevalence of AKI and in-hospital mortality had a significant difference between the generated phenogroups, phenogroup 1 was intuitively labeled as “low-risk” and phenogroup 2 as “high-risk.” We further investigated whether the generated phenogroup index could serve as an essential risk indicator for clinical outcomes of interest.

**Figure 2:**
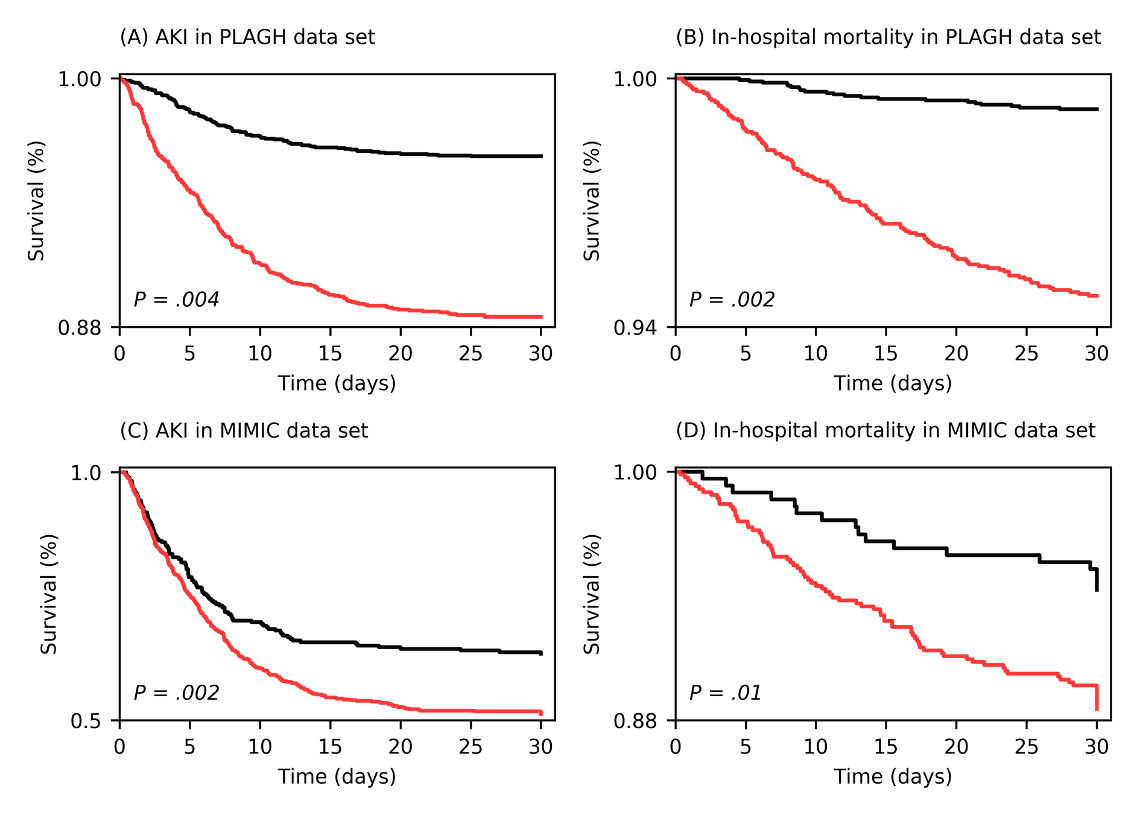
Kaplan-Meier curves for AKI and in-hospital mortality in the development (PLAGH) and external validation (MIMIC-III) data sets. AKI: acute kidney injury; MIMIC: Medical Information Mart for Intensive Care; PLAGH: PLA General Hospital.

Figure 2 shows the survival difference with respect to AKI and in-hospital mortality between the generated “high-risk” and “low-risk” phenogroups from both the PLAGH data set and the external validation MIMIC-III data set. For AKI, the curves of phenogroup 2 were lower than the curves of phenogroup 1 in both development and external validation data sets (PLAGH: *P*=.004; MIMIC-III: *P*=.002). In addition, we found that most AKI events often happened in the first few days of hospitalization in both the PLAGH and MIMIC-III data sets. This finding was in line with the literature [7,8]. For in-hospital mortality, the curves of phenogroup 2 were consistently lower than the curves of phenogroup 1 (PLAGH: *P*=.002; MIMIC-III: *P*=.01). In consideration of the baseline difference between the PLAGH data set and MIMIC-III data set, the results demonstrated that our model was robust in discriminating between high-risk and low-risk patients and easily transferable to different clinical settings.

### Outcome Prediction

Table 3 compares the prediction performances of the 5 LR models. Sensitivity, specificity, and concordance statistics are reported for the prediction performance evaluation. As the false-negative prediction (ie, neglecting AKI) may lead to extremely negative consequences, we mainly compared the sensitivity performance among the 5 models. The threshold of sensitivity and specificity was 0.5 in all experiments, and the selected top-10 variables are listed in Table S6, Multimedia Appendix 1. The results showed that the phenogroup index was an essential risk predictor of outcomes. For one, Model 1 used 1 variable (the phenogroup index) as the predictor and achieved promising sensitivity in terms of AKI (0.710) and in-hospital mortality (0.820) among the 5 prediction models with the PLAGH data set. For another, the prediction performance of Model 1 remained quite stable in the external validation (AKI sensitivity 0.760; in-hospital mortality sensitivity 0.826), while there existed significant degradation of performance in the other prediction models.

**Table 3:** Prediction performance comparison.

Model by task		PLAGH^a^ data set (development)				MIMIC-III^b^ data set (validation)		
		Sensitivity	Specificity	C-statistic^c^	Sensitivity		Specificity	C-statistics
**AKI^d^**								
	Model 1	0.710	0.577	0.643	0.760		0.342	0.551
	Model 2	0.647	0.638	0.696	0.374		0.652	0.532
	Model 3	0.679	0.723	0.756	0.478		0.562	0.546
	Model 4	0.737	0.753	0.815	0.544		0.560	0.570
	Model 5	0.718	0.746	0.816	0.573		0.540	0.575
**In-hospital mortality**								
	Model 1	0.849	0.568	0.708	0.826		0.309	0.568
	Model 2	0.791	0.736	0.824	0.530		0.672	0.622
	Model 3	0.820	0.763	0.856	0.622		0.599	0.647
	Model 4	0.835	0.809	0.899	0.490		0.746	0.646
	Model 5	0.856	0.812	0.900	0.620		0.720	0.644

^a^PLAGH: PLA General Hospital.

^b^MIMIC-III: Medical Information Mart for Intensive Care III.

^c^C-statistic: concordance statistic.

^d^AKI: acute kidney injury.

### HR Comparison

**Figure 3:**
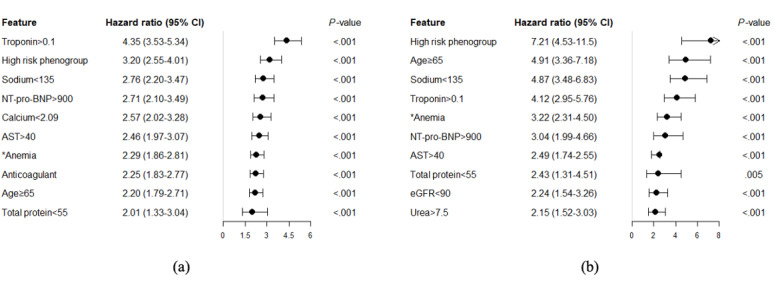
Hazard ratios of of top-ranked 10 discriminative features for (a) acute kidney injury and (b) in-hospital mortality from the PLA General Hospital data set. AST: aspartate aminotransferase; eGFR: estimated glomerular filtration rate; NT-pro-BNP: N-terminal probrain natriuretic peptide. *Anemia was defined as hemoglobin <135 g/L for men and hemoglobin <120 g/L for women. All units of variables in this figure are same as the units in Table 2.

**Figure 4:**
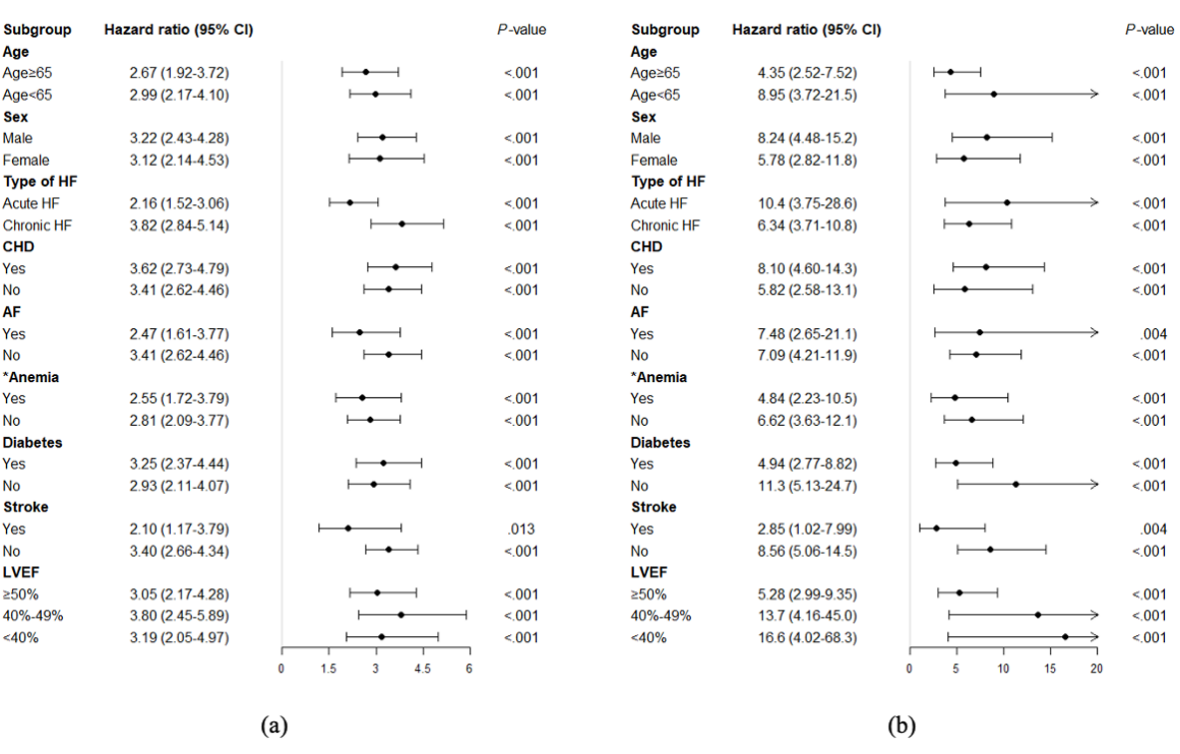
Subgroup analysis of the generated phenogroup index for (a) acute kidney injury and (b) in-hospital mortality. AF: atrial fibrillation; CHD: chronic heart disease; HF: heart failure; LVEF: left ventricular ejection fraction. *Anemia was defined as hemoglobin <135 g/L for men and hemoglobin <120 g/L for women. All units of variables in this figure are same as the units in Table 2.

We used unadjusted Cox proportional hazard regression to determine whether the phenogroup index can act as an essential risk stratification indicator in comparison with the original 39 included variables. The top-ranked 10 variables with the highest HR are listed in Figure 3 (full list is available from Figure S3, Multimedia Appendix 1). The results showed that the HR of the phenogroup index was ranked second in AKI analysis and first in in-hospital mortality analysis, indicating that the phenogroup index can be an effective risk stratification indicator compared with the original variables. Of further note, although troponin T was ranked first for AKI analysis, it was not appropriate for univariate risk indicators since only 16.73% (849/5075) of patients in the PLAGH data set had abnormal records in troponin T. Using troponin T as the indicator only achieved a sensitivity of 0.431, which was significantly lower than the performance of the phenogroup index (0.710). The association between the generated phenogroup index and risk of AKI (in-hospital mortality) was consistent in all examined subgroups (Figure 4).

## Discussion

### Principal Findings

We explored the potential of using a large volume of EHR data to cluster patients with HF and identify those with normal renal function but susceptible to de novo AKI via an unsupervised machine learning model. The experimental results showed that there was significant difference in AKI and in-hospital mortality occurrence between the 2 phenogroups generated from EHR data. As EHR is a real-world, readily available data source containing rich medical information of thousands of patients, our study demonstrated that it was possible for researchers to answer important clinical and scientific questions effectively by exploiting the huge potential of EHR data via machine learning techniques at a fraction of the resource cost that would have been required using traditional approaches [22,23].

We demonstrated that HF patients with normal renal function can be naturally separated into a “high-risk phenogroup,” of patients susceptible to de novo AKI and a “low-risk phenogroup” who were not. Patients in high-risk phenogroup were typically older, more susceptible to multi-organ dysfunction and anemia, and had significantly higher in-hospital mortality than did those in the low-risk phenogroup. These findings were in line with recent studies [17,24] and warrant further assessment. We found that patients in the high-risk phenogroup had lower levels of lipid and BMI than did those in the low-risk group. These findings are consistent with previous studies reporting that worse cardiac function may cause malnutrition [25] and a decrease of lipid level [26]. Of note, worse cardiac function was also associated with hemodynamic instability, which influences the choice of oral medication strategies [27]. We observed that patients in the high-risk phenogroup received less medication (angiotensin-converting enzyme inhibitor, angiotensin receptor blocker, calcium channel blocker, and beta blockers) than did those in the low-risk phenogroup. On the contrary, we found that patients in low-risk phenogroup were likely to receive percutaneous coronary intervention (PCI) during their stay at the emergency care unit or in hospitalization to revascularize the stable hemodynamic level such that the perfusion of the kidney could be improved and the risk of AKI significantly alleviated. This finding is consistent with previous findings, emphasizing the benefit of timely revascularization [28].

Identification of patients with HF with normal renal function but at high-risk of de novo AKI is a major challenge in HF treatment management. Clinicians have highlighted the need for more effective methods to perform this important clinical task [29]. In this study, we illustrated that machine learning analysis can tackle this challenge by providing deep integration of the comprehensive clinical variables routinely documented in EHR data. As observed in the present study, the phenogroup index generated by an unsupervised machine learning approach, as a latent representation of 39 original variables and their interactions, exhibited a sensitivity of 0.710 and 0.760 on the development data set (PLAGH) and the external validation data set (MIMIC-III). In this sense, the generated phenogroups from raw EHR data are meaningful and can be translated into actionable information for clinical decision-making. On the contrary, all other LR models met a serious overfitting problem due to the fact that the included variables had different distributions between the development (PLAGH) and external validation (MIMIC-III) data sets (as can be seen in Table S3, Multimedia Appendix 1). Inevitably, this issue caused a significant performance degeneration in the external validation. In consideration of the baseline difference between the PLAGH data set and the MIMIC-III data set, the results suggested that the generated phenogroup index was able to act as an essential de novo AKI risk indicator for patients with HF and normal renal function and be smoothly applied in different clinical settings and in different patient populations. In fact, machine learning algorithms can handle a large volume of variables and a vast number of variable-variable interactions in each patient. This merit effectively individualizes risk assessment and remedies many of the limitations of standard statistical models [22].

Our study has potentially important clinical ramifications. For one, as AKI risk is often underestimated or neglected in patient with HF, especially those with normal renal function [5], our study provided a new perspective for identifying patients with HF and normal renal function but who are at high risk of AKI. For another, in comparison with recent studies that focused on finding new biomarkers for AKI prediction or detection [30], we adopted an improved alternative strategy that used machine learning techniques to explore readily available clinical data to identify patients with HF at high risk of de novo AKI. Such meaningful use of EHR data may provide the best available evidence to assist clinical decision-making. It should be noted that these improvements may be enhanced by mining a large volume of readily available EHR data, which in turn may provide a new avenue for improving any given machine learning algorithm.

### Limitations

Several limitations of this study should be acknowledged. First, this is a single-institution study. Although we have evaluated our model on an external validation data set extracted from MIMIC-III, the methods may perform less well in other situations due to the lack of sufficient external validation samples collected from different medical facilities and in different clinical settings. Second, our study was limited by its retrospective design, and all analyses were purely observational. Although we found that there were distinct variables associated with increased risks of de novo AKI and in-hospital mortality, these nonrandomized comparisons should be interpreted cautiously in this context, and the prognostic ability of our model needs to be supported by validation in prospective studies. Third, considering the sensitivity and the specificity for AKI forecasting, our model was relatively sensitive but not very specific. Despite the influence of false-positive classification being limited in this study, further study will be required to enable machine learning–based analysis to capture the salient features distinguishing high- from low-risk cases, such that the prediction performance of our model can be improved.

### Conclusions

This study demonstrated that unsupervised machine learning–based EHR analysis is able to separate patients with HF and normal renal function into mutually exclusive phenogroups that correspond to saliently distinct AKI risk levels. Our investigation paves the way for developing an easy-to-use, broadly available model that allows the identification of patients with HF at high-risk of de novo AKI and may help improve outcomes in HF, offering a crucial advantage over traditional techniques for patient phenogrouping and clinical risk stratification.

## Appendix

Multimedia Appendix 1 Experimental data set introduction and detailed experiment results.

## Abbreviations

AF: atrial fibrillation
AKI: acute kidney injury
CHD: coronary heart disease
CKD: chronic kidney disease
CKD-EPI: Chronic Kidney Disease Epidemiology Collaboration
eGFR: estimated glomerular filtration rate
EHR: electronic health record
HF: heart failure
HR: hazard ratio
KDIGO: Kidney Disease: Improving Global Outcomes
LR: logistic regression
MIMIC: Medical Information Mart for Intensive Care
PLAGH: Chinese PLA General Hospital
SCr: serum creatinine
