# Supplementary material for: Identifying Patients With Heart Failure Who Are Susceptible to De Novo Acute Kidney Injury: Machine Learning Approach
Source: JMIR Med Inform. 2022 Oct 14;10(10):e37484. doi: 10.2196/37484 (PMC9617187; doi:10.2196/37484)
Supplement: Multimedia Appendix 1 [file medinform_v10i10e37484_app1.doc]

# Supplementary Files

**Supplementary Figure Content**

[eFigure 1 The PLAGH dataset preprocessing procedure. 1](#__RefHeading___Toc46581549)

[eFigure 2 Detailed data preprocessing procedure of MIMIC dataset. 2](#__RefHeading___Toc46581550)

[eFigure 3 Trend of Silhouette coefficient with the different cluster number. 3](#__RefHeading___Toc46581551)

[eFigure 4 T-SNE visualization of the generated phenogroups from the PLAGH Dataset. 4](#__RefHeading___Toc46581552)

[eFigure 5 HR of all features of (A) AKI and (B) in-hospital mortality, achieved from the PLAGH dataset. 5](#__RefHeading___Toc46581553)

[eFigure 6 Subgroup analysis of the phenogroup index generated for (a) AKI and (b) in-hospital mortality. 6](#__RefHeading___Toc46581554)

**Supplementary Table Content**

[eTable 1 Data Transformation Method for Continuous Variables 7](#__RefHeading___Toc46581558)

[eTable 2 Abnormal threshold of continuous variables 9](#__RefHeading___Toc46581559)

[eTable 3 Baseline Comparison between the development (PLAGH) and external validation (MIMIC) datasets 10](#__RefHeading___Toc46581560)

[eTable 4 Centroids of the generated phenogroups from PLAGH dataset 11](#__RefHeading___Toc46581561)

[eTable 5 Full baseline characteristics of PLAGH dataset and phenogroups 12](#__RefHeading___Toc46581562)

[eTable 6 Selected variables in outcome prediction 14](#__RefHeading___Toc46581563)


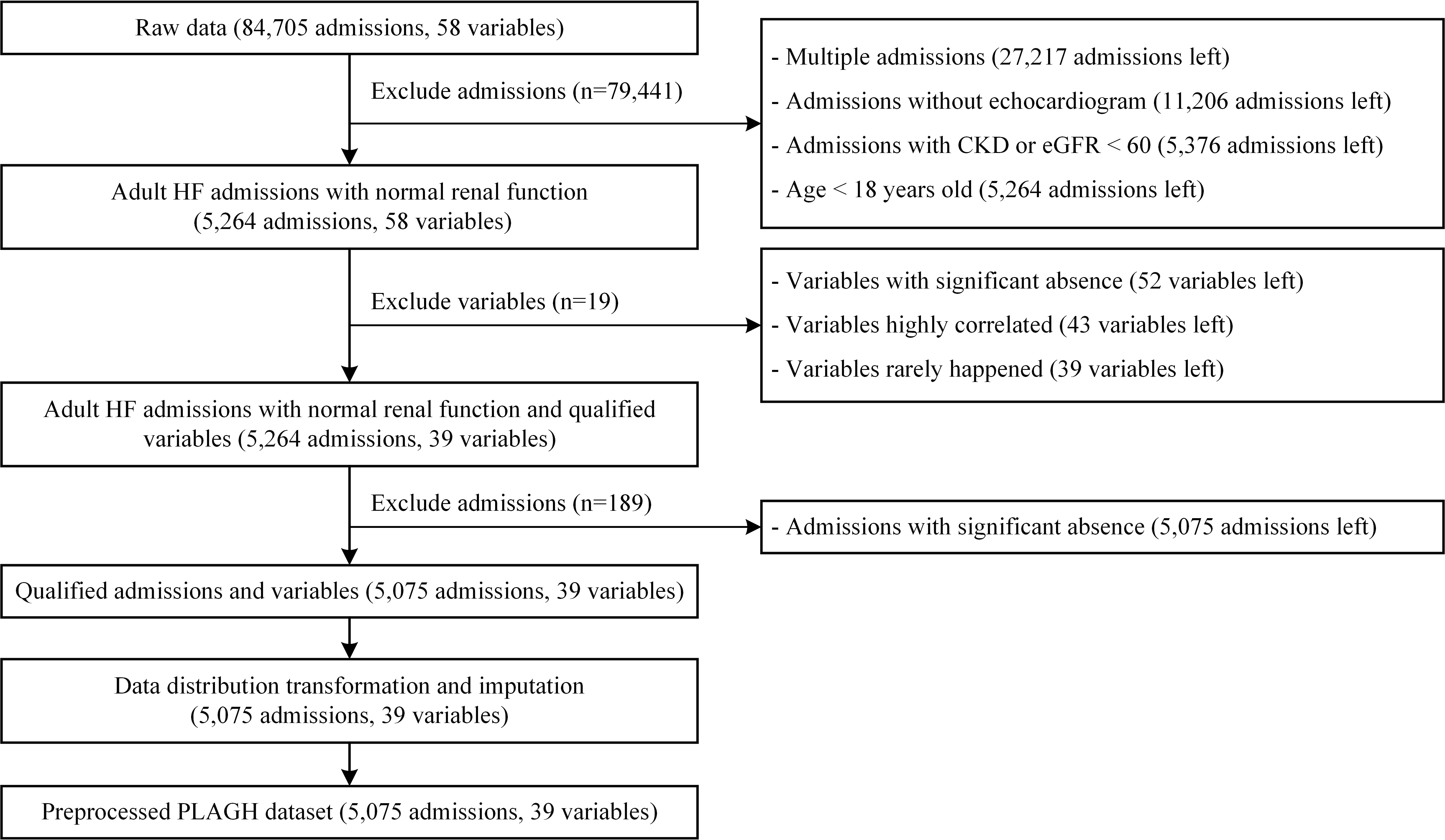


eFigure 1 The PLAGH dataset preprocessing procedure.


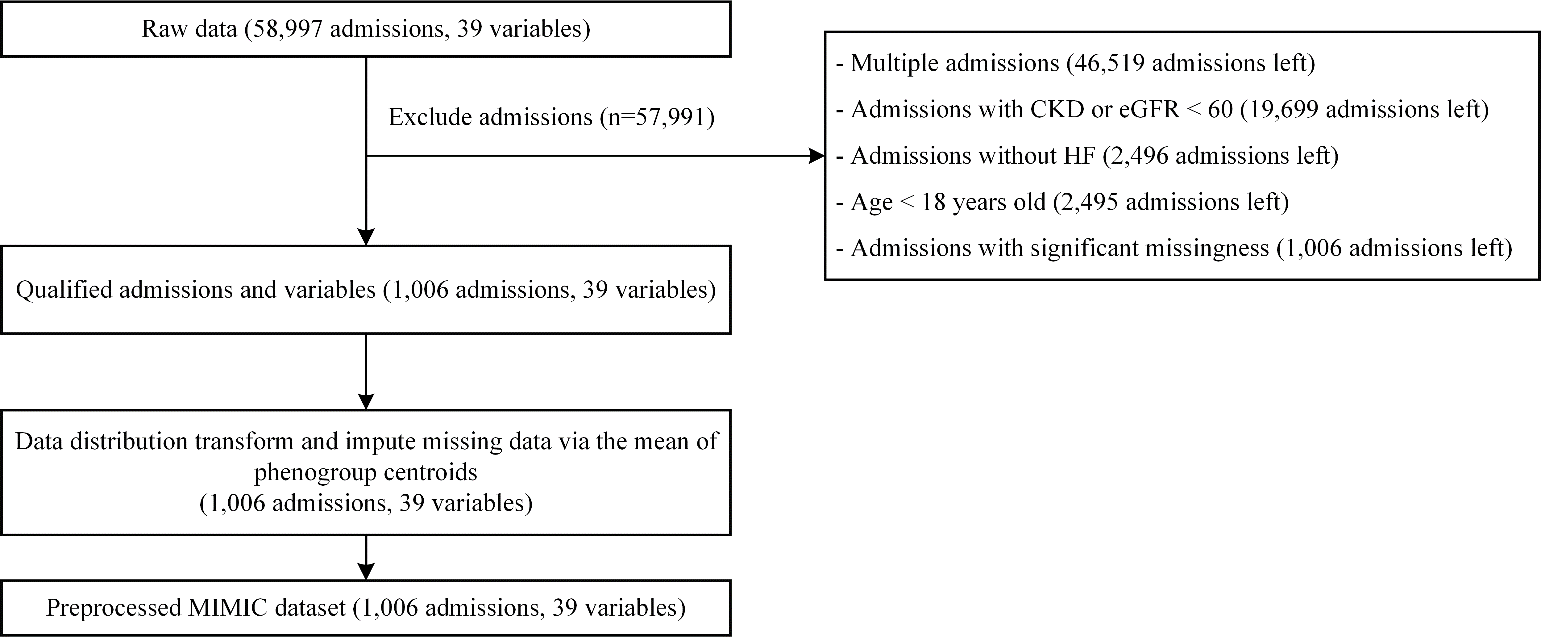


eFigure 2 Detailed data preprocessing procedure of MIMIC dataset.


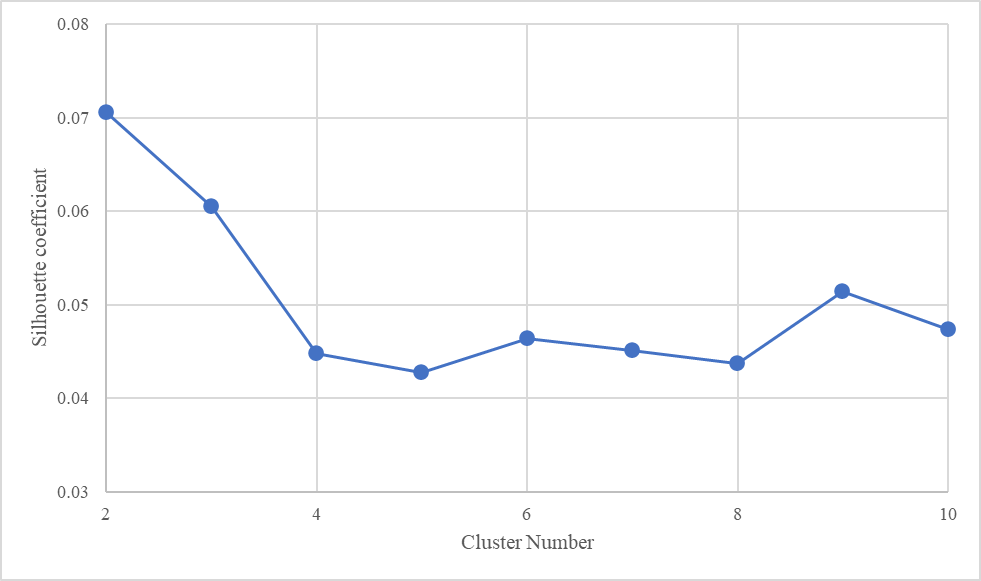


eFigure 3 Trend of Silhouette coefficient with the different cluster number.

The larger the value of Silhouette coefficient, the better the clustering performance. We tested silhouette coefficient when the cluster number ranges from 2 to 10, and set the cluster number as two, which was corresponding with the largest silhouette coefficient.


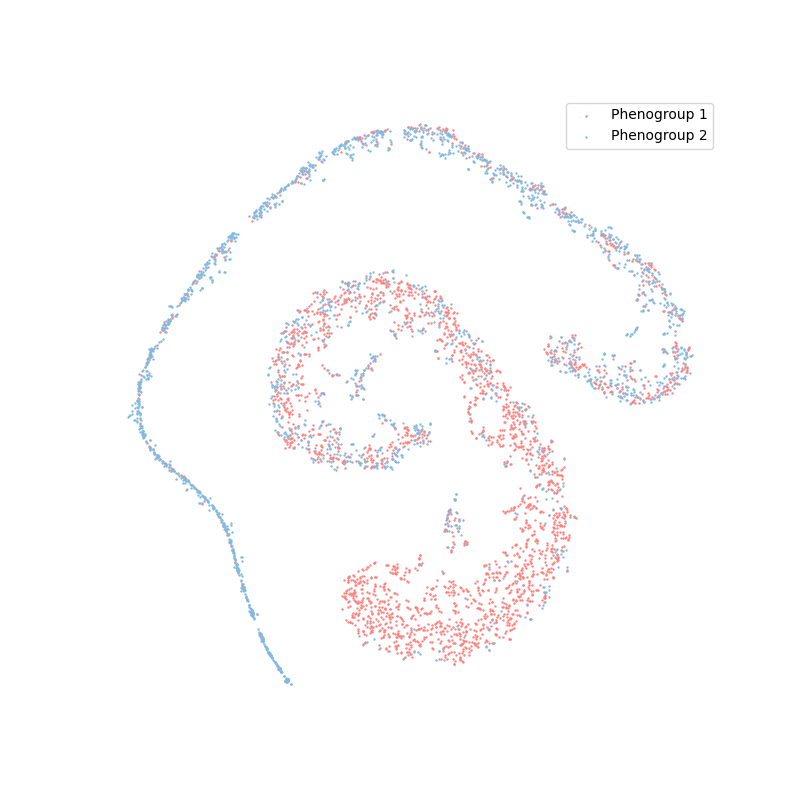


eFigure 4 T-SNE visualization of the generated phenogroups from the PLAGH Dataset.

The two phenogroups were basically projected into non-overlap areas.


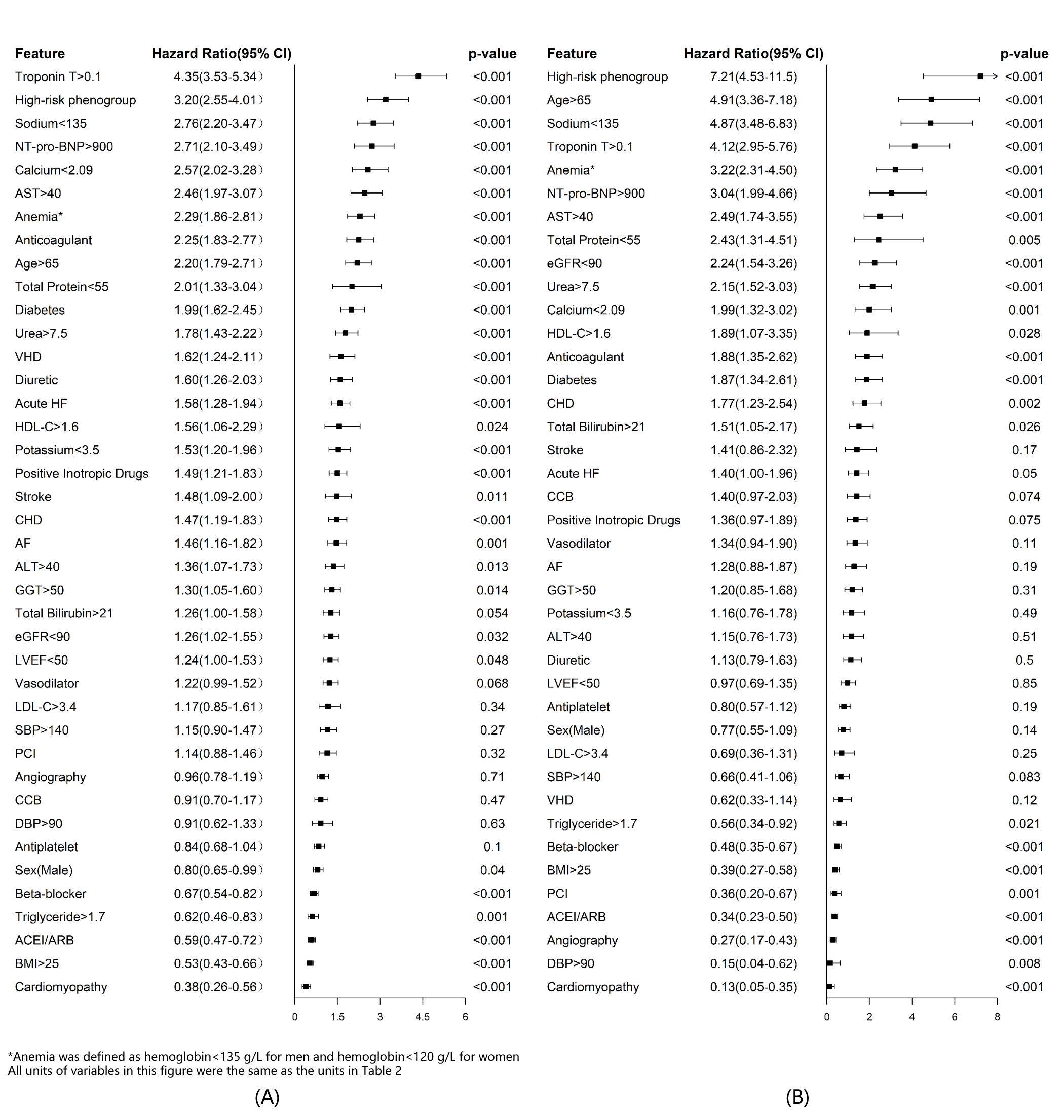


eFigure 5 HR of all features of (A) AKI and (B) in-hospital mortality, achieved from the PLAGH dataset.


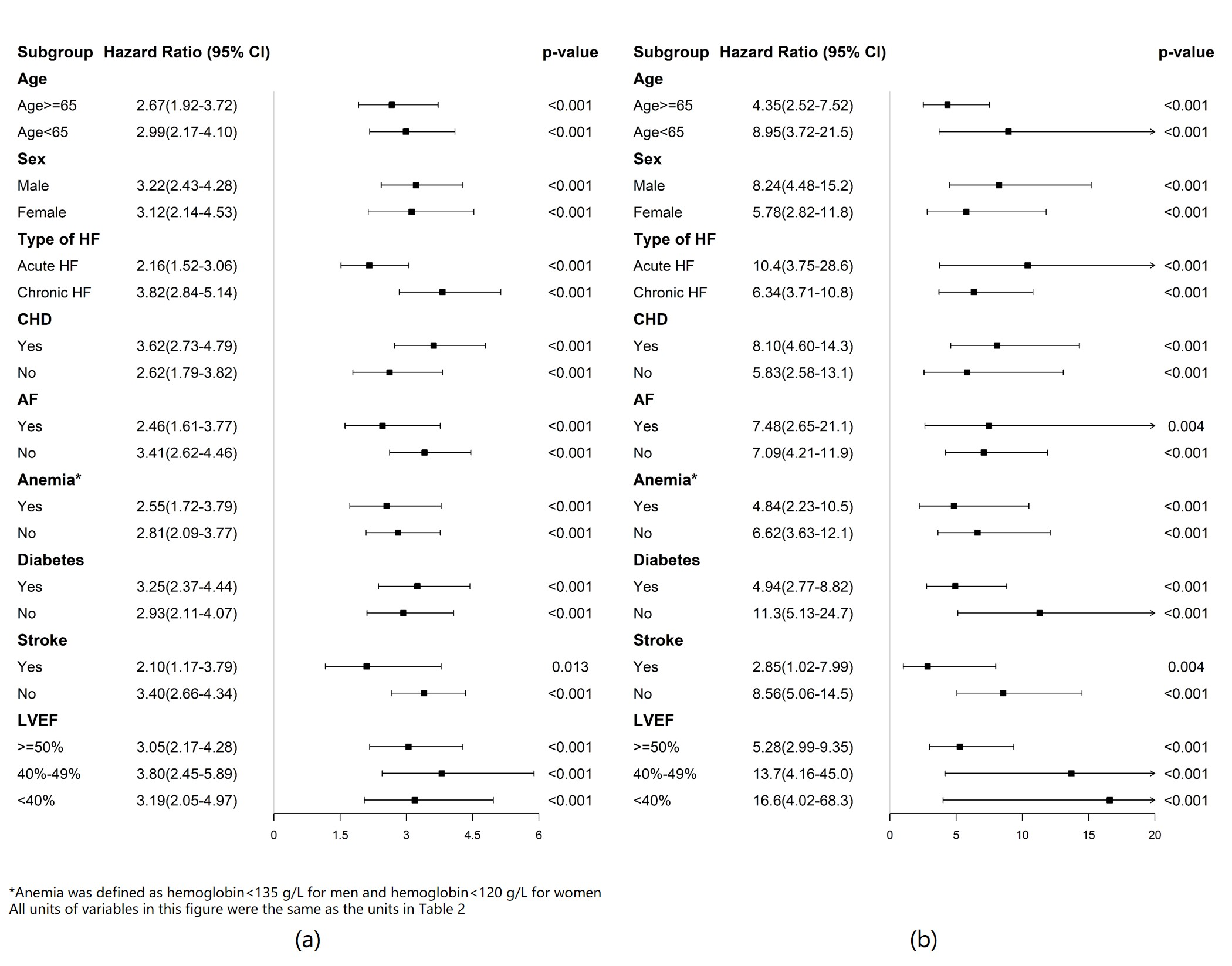


eFigure 6 Subgroup analysis of the phenogroup index generated for (a) AKI and (b) in-hospital mortality.

eTable 1 Data Transformation Method for Continuous Variables

| **Feature** | **Transform** | **Min** | **Max** | **Mean** | **Stdev** |
| --- | --- | --- | --- | --- | --- |
| Age, year | 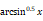 | 18.00 | 92.00 | 0.77 | 0.18 |
| ALT, IU/L | 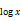 | 3.50 | 424.50 | -3.17 | 1.17 |
| AST, IU/L | 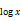 | 7.90 | 578.10 | -3.66 | 1.23 |
| BMI, kg/m^2^ | skip | 15.24 | 36.25 | 0.47 | 0.19 |
| Calcium, mmol/L | skip | 1.70 | 2.66 | 0.57 | 0.14 |
| DBP, mmHg | skip | 46.00 | 110.00 | 0.45 | 0.20 |
| eGFR, mL/min/1.73 m^2^ | 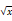 | 60.00 | 136.08 | 0.58 | 0.19 |
| GGT, IU/L | 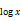 | 8.30 | 441.40 | -2.83 | 1.46 |
| HDL-C, mmol/L | skip | 0.32 | 2.11 | 0.41 | 0.18 |
| Hemoglobin, g/L | 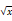 | 61.00 | 179.00 | 0.79 | 0.12 |
| LDL-C, mmol/L | skip | 0.66 | 5.05 | 0.39 | 0.19 |
| LVEF, % | skip | 20.00 | 68.00 | 0.54 | 0.27 |
| NT-pro-BNP, pg/mL | 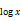 | 17.82 | 35000.00 | -3.53 | 1.99 |
| Potassium, mmol/L | skip | 2.81 | 5.75 | 0.37 | 0.15 |
| SBP, mmHg | skip | 84.00 | 190.00 | 0.40 | 0.19 |
| Sodium, mmol/L | 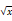 | 124.90 | 149.20 | 0.78 | 0.13 |
| Total protein, g/L | skip | 44.40 | 83.90 | 0.58 | 0.17 |
| Triglyceride, mmol/L | 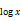 | 0.42 | 5.62 | -2.09 | 1.39 |
| Troponin T, ng/mL | 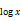 | 0.00 | 7.71 | -5.77 | 2.33 |
| Total bilirubin, μmol/L | 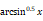 | 3.10 | 88.00 | 0.38 | 0.17 |
| Urea, mmol/L | 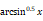 | 2.50 | 25.30 | 0.39 | 0.12 |

1. “Skip” denotes the variable following normal distribution originally, and thus we can directly transform these variables into normal distribution without using a numerical transform function.
2. We used the 0.01-fractile (the “Min” column in the table) and 0.99-fractile (the “Max” column in the table) of a variable as the cut-off point to eliminate the error value or harmful outliers in advance.
3. The “Mean” and “Stdev” indicate the mean and standard deviation of variable transformed by the “Transform” function.

For a continuous variable
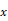
, we used the formula:


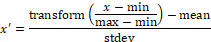


to transform it into a new variable that follows standard normal distribution.

For example, if we have a patient whose age is 85, the transformed value can be calculated as:


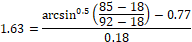


eTable 2 Abnormal threshold of continuous variables

| **Feature** | **Upper Threshold** | **Lower Threshold** |
| --- | --- | --- |
| Age, year | 65 | / |
| ALT, IU/L | 40 | / |
| AST, IU/L | 40 | / |
| BMI, kg/m^2^ | 25 | / |
| DBP, mmHg | 90 | / |
| Calcium, mmol/L | / | 2.09 |
| eGFR, mL/min/1.73m^2^ | / | 90 |
| Glucose, mmol/L | 7 | / |
| GGT, IU/L | 50 | / |
| Hemoglobin, g/L | / | 135 for men and 120 for women |
| HDL-C, mmol/L | / | 1.0 |
| LDL-C, mmol/L | 3.4 | / |
| LVEF, % * | / | 40 |
| NT-pro-BNP, pg/mL | 900 | / |
| Potassium, mmol/L | / | 3.5 |
| SBP, mmHg | 140 | / |
| Sodium, mmol/L | / | 135 |
| Total bilirubin, μmol/L | 21 | / |
| Total protein, g/L | / | 55 |
| Triglyceride, mmol/L | 1.7 | / |
| Troponin T, ng/mL | 0.1 | / |
| Urea, mmol/L | 7.1 | / |

1. Variables less than the lower threshold or larger than the upper threshold will be recorded as abnormal record. The threshold was obtained mainly in the EHR system of PLAGH.

eTable 3 Baseline Comparison between the development (PLAGH) and external validation (MIMIC) datasets

| **Feature** | **MIMIC Dataset (1,006)** | | **PLAGH Dataset (5,075)** | **p-value** |
| --- | --- | --- | --- | --- |
| AKI | 0.451 (454) | | 0.071 (365) | <0.001 |
| In-hospital Mortality | 0.097 (98) | | 0.027 (139) | <0.001 |
| ACEI | 0.41 (415) | | 0.36 (1831) | 0.002 |
| Acute HF | 0.34 (1723) | | 0.25 (247) | <0.001 |
| AF | 0.32 (331) | | 0.22 (1121) | <0.001 |
| Age, year | 66.51 (56.56-77.19) | | 60.99 (51.00-70.00) | <0.001 |
| ALT, IU/L | 29.00 (18.00-50.00) | | 21.00 (14.39-33.79) | <0.001 |
| Angiography | 0.49 (496) | | 0.39 (2008) | <0.001 |
| Anticoagulant | 0.76 (773) | | 0.37 (1927) | <0.001 |
| Antiplatelet | 0.63 (642) | | 0.61 (3103) | 0.479 |
| ARB | 0.04 (41) | | 0.15 (808) | <0.001 |
| AST, IU/L | 35.00 (22.00-66.00) | | 21.29 (16.29-30.40) | <0.001 |
| Beta-blocker | 0.70 (707) | | 0.67 (3428) | 0.090 |
| BMI, kg/m^2^ | 25.02 (24.73-29.83) | | 24.60 (22.45-27.09) | <0.001 |
| Calcium, mmol/L | 2.15 (2.02-2.27) | | 2.24 (2.16-2.33) | <0.001 |
| Cardiomyopathy | 0.12 (122) | | 0.18 (941) | <0.001 |
| CCB | 0.15 (154) | | 0.21 (1110) | <0.001 |
| CHD | 0.67 (682) | | 0.57 (2928) | <0.001 |
| DBP, mmHg | 65 (55-74) | | 74 (67-81) | <0.001 |
| Diuretic | 0.55 (561) | | 0.67 (3407) | <0.001 |
| eGFR, mL/min/1.73m^2^ | 83.88 (71.48-94.90) | | 87.62 (75.65-98.80) | <0.001 |
| GGT, IU/L | 55.10 (55.10-55.10) | | 34.80 (21.90-63.79) | <0.001 |
| Glucose, mmol/L | 7.44 (6.05-9.50) | | 5.76 (4.86-7.57) | <0.001 |
| Hemoglobin, g/L | 125 (110-140) | | 137.00 (124-150) | <0.001 |
| HDL-C, mmol/L | 1.05 (1.05-1.08) | | 1.03 (0.85-1.22) | <0.001 |
| LDL-C, mmol/L | 2.33 (2.30-2.33) | | 2.25 (1.79-2.80) | 0.597 |
| LVEF, % * | 45.60 (45.60-45.60) | | 46.00 (35.00-56.00) | 0.643 |
| NT-pro-BNP, pg/mL* | 2651.44 (2651.44-2651.44) | | 1214 (422-2956) | 0.436 |
| PCI | 0.20 (205) | | 0.19 (969) | 0.345 |
| Positive inotropic drugs | 0.18 (191) | | 0.36 (1867) | <0.001 |
| Potassium, mmol/L | 4.10 (3.80-4.40) | | 3.89 (3.62-4.17) | <0.001 |
| SBP, mmHg | 121 (106-136) | | 124 (113-138) | <0.001 |
| Sex, male | 0.59 (597) | | 0.67 (3431) | <0.001 |
| Sodium, mmol/L | 138.00 (136.00-141.00) | | 140.70 (138.10-142.70) | <0.001 |
| Stroke | 0.05 (59) | | 0.09 (485) | <0.001 |
| Urea, mmol/L | 6.06 (4.64-7.85) | | 5.84 (4.73-7.25) | <0.001 |
| Total bilirubin, μmol/L | 10.26 (6.84-17.10) | | 13.69 (9.80-19.90) | <0.001 |
| Total Protein, g/L | 67.25 (67.25-67.25) | | 67.50 (63.30-71.80) | <0.001 |
| Triglyceride, mmol/L | 1.32 (1.17-1.37) | | 1.11 (0.83-1.59) | <0.001 |
| Troponin T, ng/mL | 0.21 (0.01-0.30) | | 0.01 (0.01-0.04) | <0.001 |
| Vasodilator | 0.30 (302) | | 0.58 (2989) | <0.001 |
| VHD | 0.24 (249) | 0.12 (616) | | <0.001 |

1. *, most NT-pro-BNP and LVEF values in MIMIC dataset were imputed.

eTable 4 Centroids of the generated phenogroups from PLAGH dataset

| **Feature** | **Phenogroup 1 Centroids**  **Origin Value / Transformed Value** | **Phenogroup 2 Centroids**  **Origin Value / Transformed Value** |
| --- | --- | --- |
| ACEI/ARB | 0.542/0.542 | 0.451/0.451 |
| Acute HF | 0.261/0.261 | 0.437/0.437 |
| AF | 0.186/0.186 | 0.264/0.264 |
| Age, year | 56.04/-0.19 | 63.54/0.250 |
| ALT, IU/L | 20.69/-0.02 | 21.92/0.032 |
| Angiography | 0.464/0.464 | 0.309/0.309 |
| Anticoagulant | 0.350/0.350 | 0.416/0.416 |
| Antiplatelet | 0.678/0.678 | 0.614/0.614 |
| AST, IU/L | 19.78/-0.17 | 26.97/0.214 |
| Beta-blocker | 0.701/0.701 | 0.642/0.642 |
| BMI, kg/m^2^ | 26.21/0.292 | 23.15/-0.46 |
| Calcium, mmol/L | 2.287/0.328 | 2.185/-0.41 |
| Cardiomyopathy | 0.176/0.176 | 0.197/0.197 |
| CCB | 0.279/0.279 | 0.142/0.142 |
| CHD | 0.588/0.588 | 0.563/0.563 |
| DBP, mmHg | 77.84/0.235 | 70.84/-0.32 |
| Diuretic | 0.569/0.569 | 0.798/0.798 |
| eGFR, mL/min/1.73m^2^ | 89.46/0.239 | 80.42/-0.29 |
| GGT, IU/L | 31.04/-0.07 | 37.38/0.091 |
| Glucose, mmol/L | 0.368/0.368 | 0.426/0.426 |
| Hemoglobin, g/L | 141.8/0.321 | 125.3/-0.39 |
| HDL-C, mmol/L | 1.069/0.044 | 1.038/-0.05 |
| LDL-C, mmol/L | 2.549/0.216 | 2.100/-0.31 |
| LVEF, % | 48.33/0.196 | 42.56/-0.24 |
| NT-pro-BNP, pg/mL | 462.5/-0.41 | 2582./0.460 |
| PCI | 0.219/0.219 | 0.154/0.154 |
| Positive inotropic drugs | 0.275/0.275 | 0.483/0.483 |
| Potassium, mmol/L | 3.879/-0.03 | 3.913/0.044 |
| SBP, mmHg | 132.0/0.276 | 118.5/-0.38 |
| Sex, male | 0.688/0.688 | 0.660/0.660 |
| Sodium, mmol/L | 141.0/0.264 | 138.0/-0.33 |
| Stroke | 0.089/0.089 | 0.103/0.103 |
| Total bilirubin, μmol/L | 13.57/-0.19 | 18.38/0.243 |
| Total protein, g/L | 69.56/0.317 | 64.78/-0.39 |
| Triglyceride, mmol/L | 1.311/0.238 | 0.813/-0.35 |
| Troponin T, ng/mL | 0.014/-0.32 | 0.047/0.259 |
| Urea, mmol/L | 5.420/-0.22 | 6.529/0.285 |
| Vasodilator | 0.601/0.601 | 0.623/0.623 |
| VHD | 0.119/0.119 | 0.124/0.124 |

eTable 5 Full baseline characteristics of PLAGH dataset and phenogroups

| **Feature** | **Population** | **Phenogroup 1** | **Phenogroup 2** | **p-value** |
| --- | --- | --- | --- | --- |
| ACEI/ARB | 0.50 (2547) | 0.54 (1531) | 0.45 (1016) | <0.001 |
| Acute HF | 0.33 (1723) | 0.26 (738) | 0.43 (985) | <0.001 |
| AF | 0.22 (1121) | 0.18 (526) | 0.26 (595) | <0.001 |
| Age, year | 61.00 (51.00-70.00) | 58.00 (48.00-67.00) | 65.00 (55.00-75.00) | <0.001 |
| Albumin | 39.63 (36.40-42.30) | 41.30 (39.10-43.60) | 37.00 (33.80-39.70) | <0.001 |
| Alkaline phosphatase，IU/L | 68.70 (56.40-84.70) | 67.40 (55.99-81.90) | 71.50 (57.39-91.40) | <0.001 |
| ALT, IU/L | 21.00 (14.39-33.79) | 20.80 (14.70-31.99) | 21.54 (13.80-36.49) | <0.001 |
| Anemia | 0.13 (670) | 0.04 (129) | 0.24 (541) | <0.001 |
| Angiography | 0.39 (2008) | 0.46 (1311) | 0.30 (697) | <0.001 |
| Anticoagulant | 0.37 (1927) | 0.35 (989) | 0.41 (938) | <0.001 |
| Antiplatelet | 0.64 (3298) | 0.67 (1914) | 0.61 (1384) | <0.001 |
| AST, IU/L | 21.29 (16.29-30.50) | 19.60 (15.50-26.00) | 24.29 (18.09-38.80) | <0.001 |
| Beta-blocker | 0.67 (3428) | 0.70 (1981) | 0.64 (1447) | <0.001 |
| BMI, kg/m^2^ | 24.60 (22.46-27.08) | 25.88 (23.87-28.08) | 23.05 (20.95-25.01) | <0.001 |
| Calcium, mmol/L | 2.24 (2.16-2.33) | 2.28 (2.21-2.36) | 2.19 (2.10-2.27) | <0.001 |
| Cardiac resynchronization therapy with a defibrillator | 0.00 (30) | 0.00 (15) | 0.00 (15) | 0.533 |
| Cardiomyopathy | 0.18 (941) | 0.17 (497) | 0.19 (444) | 0.054 |
| CCB | 0.21 (1110) | 0.27 (789) | 0.14 (321) | <0.001 |
| CHD | 0.57 (2928) | 0.58 (1660) | 0.56 (1268) | 0.073 |
| Coronary artery bypass grafting | 0.00 (40) | 0.01 (32) | 0.00 (8) | 0.002 |
| Creatine kinase, IU/L | 68.30 (46.60-108.50) | 68.30 (48.50-102.20) | 68.45 (43.60-121.00) | 0.481 |
| DBP, mmHg | 74.00 (67.00-81.00) | 77.00 (70.00-85.00) | 70.00 (64.00-78.00) | <0.001 |
| Diabetes | 0.39 (2002) | 0.36 (1041) | 0.42 (961) | <0.001 |
| Direct bilirubin, μmol/L | 4.50 (3.09-6.89) | 4.00 (2.80-5.50) | 5.50 (3.59-9.60) | <0.001 |
| Diuretic | 0.67 (3407) | 0.56 (1608) | 0.79 (1799) | <0.001 |
| eGFR, mL/min/1.73m^2^ | 87.62 (75.65-98.80) | 92.06 (80.84-101.91) | 81.85 (70.90-92.91) | <0.001 |
| GGT, IU/L | 34.80 (21.90-63.79) | 31.70 (21.30-54.89) | 40.30 (23.09-75.00) | <0.001 |
| Glucose, mmol/L | 5.76 (4.86-7.57) | 5.63 (4.83-7.30) | 5.98 (4.93-7.96) | <0.001 |
| HDL-C, mmol/L | 1.02 (0.85-1.22) | 1.04 (0.88-1.22) | 1.01 (0.82-1.22) | <0.001 |
| Hemoglobin | 137.00 (124.00-150.00) | 143.00 (132.00-154.00) | 129.00 (116.00-142.00) | <0.001 |
| Hyperlipemia | 0.35 (1786) | 0.47 (1354) | 0.19 (432) | <0.001 |
| Hypertension | 0.44 (2257) | 0.50 (1432) | 0.36 (825) | <0.001 |
| Implantable cardioverter defibrillator | 0.00 (19) | 0.00 (11) | 0.00 (8) | 0.842 |
| Lactate dehydrogenase, IU/L | 185.60 (154.00-236.49) | 171.60 (147.00-211.90) | 207.90 (169.40-280.60) | <0.001 |
| LDL-C, mmol/L | 2.25 (1.79-2.81) | 2.46 (1.96-3.05) | 2.04 (1.62-2.48) | <0.001 |
| LVEF, % | 46.00 (35.00-56.00) | 50.00 (39.00-58.00) | 41.00 (31.00-54.00) | <0.001 |
| NT-pro-BNP, pg/mL | 1215.84 (422.40-2950.00) | 571.80 (223.69-1319.00) | 2680.77 (1355.00-5188.00) | <0.001 |
| Pacemaker | 0.01 (69) | 0.01 (39) | 0.01 (30) | 0.88 |
| PCI | 0.19 (969) | 0.21 (620) | 0.15 (349) | <0.001 |
| Positive inotropic drugs | 0.36 (1867) | 0.27 (778) | 0.48 (1089) | <0.001 |
| Potassium, mmol/L | 3.89 (3.62-4.17) | 3.87 (3.62-4.13) | 3.91 (3.61-4.20) | 0.005 |
| Red blood cell 10^9^/L | 4.49 (4.08-4.88) | 4.66 (4.31-5.02) | 4.23 (3.81-4.67) | <0.001 |
| SBP, mmHg | 124.79 (113.00-138.00) | 130.00 (119.00-143.00) | 117.59 (106.00-130.00) | <0.001 |
| Scr, μmol/L | 75.70 (65.00-87.10) | 74.29 (63.70-84.50) | 77.40 (67.10-90.29) | <0.001 |
| Sex | 0.67 (3431) | 0.68 (1943) | 0.66 (1488) | 0.037 |
| Sodium, mmol/L | 140.70 (138.10-142.70) | 141.30 (139.40-143.20) | 139.40 (136.30-142.00) | <0.001 |
| Statin | 0.58 (2989) | 0.63 (1793) | 0.53 (1196) | <0.001 |
| Stroke | 0.09 (485) | 0.08 (252) | 0.10 (233) | 0.087 |
| Total bilirubin, μmol/L | 13.69 (9.80-19.90) | 12.79 (9.40-17.40) | 15.85 (10.39-24.60) | <0.001 |
| Total cholesterol, mmol/L | 3.79 (3.22-4.50) | 4.08 (3.45-4.81) | 3.49 (2.98-4.07) | <0.001 |
| Total Protein, g/L | 67.50 (63.30-71.80) | 69.20 (65.80-73.30) | 65.10 (60.40-69.00) | <0.001 |
| Triglyceride, mmol/L | 1.11 (0.82-1.59) | 1.34 (0.98-1.87) | 0.92 (0.72-1.21) | <0.001 |
| Troponin T, ng/mL | 0.01 (0.01-0.04) | 0.01 (0.00-0.02) | 0.02 (0.01-0.10) | <0.001 |
| Urea, mmol/L | 5.84 (4.73-7.25) | 5.46 (4.51-6.60) | 6.45 (5.11-8.12) | <0.001 |
| Uric acid, μmol/L | 352.60 (284.20-434.90) | 351.10 (289.60-423.60) | 354.45 (275.00-452.40) | 0.132 |
| Urine Red Blood Cell #/hpf | 0.05 (274) | 0.03 (94) | 0.07 (180) | <0.001 |
| Vasodilator | 0.61 (3103) | 0.60 (1698) | 0.62 (1405) | 0.104 |
| VHD | 0.12 (616) | 0.11 (336) | 0.12 (280) | 0.565 |

eTable 6 Selected variables in outcome prediction

| **Models** | **Selected 10 top-ranked Variables** |
| --- | --- |
| AKI Model 2 | PCI, Age, Acute HF, Sex, Antiplatelet, Diabetes, NT-pro-BNP, Vasodilator, Angiography, CCB |
| AKI Model 3 | ACEI/ARB, PCI, eGFR, Acute HF, Sex, Anticoagulant, Antiplatelet, Troponin T, NT-pro-BNP, Angiography |
| In-hospital Mortality Model 2 | PCI, eGFR, AST, Age, Acute HF, Sex, Total bilirubin, Diabetes, Vasodilator, Angiography |
| In-hospital Mortality Model 3 | PCI, eGFR, ALT, AST, Serum urea, Age, Acute HF, Sex, Diabetes, Angiography |
